# A high-density intraspecific SNP linkage map of pigeonpea (*Cajanas cajan* L. Millsp.)

**DOI:** 10.1371/journal.pone.0179747

**Published:** 2017-06-27

**Authors:** Sheetal Arora, Ajay Kumar Mahato, Sangeeta Singh, Paritra Mandal, Shefali Bhutani, Sutapa Dutta, Giriraj Kumawat, Bikram Pratap Singh, A. K. Chaudhary, Rekha Yadav, K. Gaikwad, Amitha Mithra Sevanthi, Subhojit Datta, Ranjeet S. Raje, Tilak R. Sharma, Nagendra Kumar Singh

**Affiliations:** 1 National Research Centre on Plant Biotechnology, Pusa Campus, New Delhi, India; 2 Indian Institute of Pulses Research. Kanpur, India; 3 Division of Genetics, Indian Agricultural Research Institute, Pusa, New Delhi, India; Mahatma Phule Krishi Vidyapeeth College of Agriculture, INDIA

## Abstract

Pigeonpea (*Cajanus cajan* (L.) Millsp.) is a major food legume cultivated in semi-arid tropical regions including the Indian subcontinent, Africa, and Southeast Asia. It is an important source of protein, minerals, and vitamins for nearly 20% of the world population. Due to high carbon sequestration and drought tolerance, pigeonpea is an important crop for the development of climate resilient agriculture and nutritional security. However, pigeonpea productivity has remained low for decades because of limited genetic and genomic resources, and sparse utilization of landraces and wild pigeonpea germplasm. Here, we present a dense intraspecific linkage map of pigeonpea comprising 932 markers that span a total adjusted map length of 1,411.83 cM. The consensus map is based on three different linkage maps that incorporate a large number of single nucleotide polymorphism (SNP) markers derived from next generation sequencing data, using Illumina GoldenGate bead arrays, and genotyping with restriction site associated DNA (RAD) sequencing. The genotyping-by-sequencing enhanced the marker density but was met with limited success due to lack of common markers across the genotypes of mapping population. The integrated map has 547 bead-array SNP, 319 RAD-SNP, and 65 simple sequence repeat (SSR) marker loci. We also show here correspondence between our linkage map and published genome pseudomolecules of pigeonpea. The availability of a high-density linkage map will help improve the anchoring of the pigeonpea genome to its chromosomes and the mapping of genes and quantitative trait loci associated with useful agronomic traits.

## Introduction

Pigeonpea (*Cajanus cajan* L. Millsp.) is a diploid species (2n = 2x = 22) belonging to the genus *Cajanus* and is a member of the plant family Fabaceae [1]. It is a warm-season legume crop with a moderate genome size of 858 Mb. Pigeonpea is an important crop owing to its high nutritional value, drought resilience, disease resistance, and ability to improve soil carbon content [2]. It is particularly rich in protein, vitamins, minerals, and essential secondary metabolites like isoflavonoids; therefore, pigeonpea is significant in the remediation of undernourishment or protein-calorie malnutrition [3], which makes pigeonpea a highly promising crop for eliminating malnutrition, especially in South Asia.

Owing to various abiotic and biotic stresses and unavailability of quality seeds of improved cultivars, growth in pigeonpea yield has remained consistently low for decades. The genetics of important agronomic traits in pigeonpea have not yet been worked out and their molecular basis remains poorly understood. Furthermore, cultivated pigeonpea has a low level of DNA polymorphism in the primary gene pool and there is a lack of validated molecular markers in this species [4,5]. A Pigeonpea Genomics Initiative (PGI) was started by the Indian Council of Agricultural Research (ICAR) to address these issues under the Indo-US Agricultural Knowledge Initiative (AKI). The AKI-PGI has contributed significantly to the generation of genomic resources for pigeonpea to facilitate marker-assisted breeding (MAB) by developing numerous simple sequence repeat (SSR) and single nucleotide polymorphism (SNP) markers and moderately dense linkage maps. Draft genomes of the pigeonpea variety Asha has been assembled using FLX 454 (Roche Inc., Germany) and Illumina (San Diego, USA) sequencing technologies [6,7]; however, it has not been possible to map a large number of loci in a single mapping population owing to the low level of polymorphism. Hence, to increase the number of mapped markers and construct a dense consensus map, multiple mapping populations with an acceptable number of common markers are utilized.

Recent efforts towards building a genetic map of pigeonpea have led to the development of several interspecific and intraspecific maps. The first interspecific map of pigeonpea was developed using 554 diversity arrays technology (DArT) markers covering a total map distance of 451.6 cM [8]. Recently, another interspecific linkage map was reported with 191 start codon targeted (SCoT), inter simple sequence repeats (ISSR), and random amplified polymorphic DNA (RAPD) markers mapped onto 11 linkage groups covering a total map length of 1,624.71 cM with an average marker interval of 8.51 cM [9]. An intraspecific medium density consensus map of six different mapping populations with 339 SSR loci spanning total map length of 1,059 cM has been reported [10]. Another medium density intraspecific map from a single mapping population has been reported that comprises 296 genic SNP and SSR markers covering a total map length of 1,520.22 cM with average map interval of 4.95 cM [11]. These maps have helped QTL mapping of agronomically useful traits and anchoring of the pigeonpea draft genome.

Earlier linkage maps of pigeonpea have employed mostly DArT, RAPD, ISSR, and SSR markers. Although these are useful markers, some of these have dominant type inheritance; therefore, in the present study, we have exploited more informative co-dominantly inherited SSR and SNP markers. In the last few years, SNPs have become the markers of choice for all species owing to their abundance in the genome, high-throughput assays, and low cost per data point. In recent years, SNP genotyping by restriction site associated DNA (RAD) sequencing has been favored because it makes available a genome-wide representation of all the sites of a particular restriction enzyme at an affordable cost. Therefore, we chose SNP markers to prepare a high-density linkage map of pigeonpea comprising 932 markers. The component linkage maps were developed mainly with SNP markers using the Illumina’s GoldenGate assay and reduced representation RAD genotyping-by-sequencing (GBS) approaches [12].

## Materials and methods

### Plant material and DNA extraction

Three different intraspecific F_2_ mapping populations, Asha/UPAS 120 (population A) consisting of 92 individuals, Pusa Dwarf/H2001-4 (population R) consisting of 94 individuals, and Pusa Dwarf/HDM04-1 (population G) consisting of 186 individuals [11], were used for the construction of the pigeonpea linkage map. The seeds of original parents were obtained from ICAR-IARI Division of Genetics, New Delhi and ICAR-IIPR, Kanpur, India. Genomic DNA was isolated from the fresh young leaves of F_2_ individuals grown in net house along with their parents using a modified CTAB method [13].

### Genotyping using SSR markers

A total of 222 SSR markers including 71 genic arhar SSR (ASSR) [14] and 151 genomic highly variable arhar SSR (HASSR) [15] were used for genotyping 92 F_2_ individuals in population A. The PCR reactions were carried out in a PTC225 Gradient Cycler (MJ Research, Waltham, USA). Each PCR reaction in a final reaction volume of 15 μl consisted of 1.5 μl 10x reaction buffer, 0.20 μl 10 mM dNTPs (133 μM), 1.5 μl each forward and reverse primers (10 pmol each), and 2.0 μl (70 ng) template genomic DNA, 0.15 μl (0.75 U) Taq DNA polymerase (Vivantis Technologies, CA, USA). The PCR thermal profile included an initial denaturation at 94°C for 5 min. followed by 35 cycles of 94°C for 1 min., 55°C for 1 min., and 72°C for 1 min., and finally, 10 min at 72°C for a final extension. The PCR products were resolved by electrophoresis in 4% metaphor agarose gels or 8% polyacrylamide gels. After electrophoresis, PCR products were visualized and photographed in a Fluorchem™ 5500 (Alfa Innotech Crop, USA) gel documentation system. In population G, of the 595 SSR markers screened for parental polymorphism between Pusa Dwarf/HDM04-1, only 29 (4.8%) were found to be polymorphic [11].

### SNP genotyping using GoldenGate assays

Two different multiplexed SNP assays using 768 and 1,536 genic SNP markers were used, employing Illumina GoldenGate bead-array technology (http://www.illumina.com), as described earlier [11]. The 1,536-plex assay was used for genotyping of all three pigeonpea populations (A, G and R), whereas the 768-plex assay was used for the populations A and G only.

### SNP genotyping by RAD sequencing

RAD sequencing was performed on two sets of 96-plex libraries using restriction enzymes *Apek*I and *Pst*I on 92 F_2_ individuals and their parents using the raw sequence reads that were generated for the optimized RAD library with Illumina True-Seq V3 paired-end chemistry and average read lengths of 100 bp on an Illumina HiSeq platform. Bioinformatics analysis included selecting high quality reads from the raw data at a threshold quality score of Q20 and the removal of adapter sequences, mapping high quality reads, and SNP detection from GBS data with TASSEL version 3.0 [16]. The reference sequence used in this analysis covered approximately 60% of the pigeonpea genome. In the TASSEL software, the reads were sorted after alignment and identical reads were collapsed into a single tag. Default parameters were used for the previous steps, parameters used during SNPs calling included: inbreeding coefficient (mnf) = -1, minimum minor allele frequency (mnMAF) = 0.1, minimum locus coverage (mnLCov) = 0.05, minimum site coverage (mnSCov) = 0.70, minimum taxon coverage (mnTCov) = 0.50, and the “hLD” option was invoked once for each of the two enzymes. TASSEL version 4.0 was used for the integration of data from both runs, once for each enzyme to obtain a single HapMap output file.

### Construction of individual and consensus linkage maps

The F_2_ mapping population A of Asha/UPAS 120 was used to generate the genetic reference map for pigeonpea. Genotyping data was generated from 92 F_2_ individuals for 10,786 polymorphic marker loci using different marker systems that included Illumina GoldenGate genic SNP, RAD-GBS SNP, ASSR, and HASSR markers. JoinMap 4.0 [17] was used for linkage map construction. A chi-square test was performed on the genotyping data for this population to test the goodness of fit with the expected segregation ratio of 1:2:1. To include more markers in the map, the cut-off chi-square value was relaxed to 13.5. The grouping of markers was done on the basis of cut off Rf value of 0.25 and a logarithm of the odds (LOD) score of 3.0. In the F_2_ mapping population R (Pusa Dwarf/H2001-4), the chi-square cut-off was set at 9.2 and all markers followed the expected segregation ratio of 1:2:1. The linkage map of Pusa Dwarf/HDM04-1 (population G) was previously drawn using MapDisto software [18] and has been used for mapping QTLs for plant height, stem branching, and maturity time [11]. This map was redrawn in this study using JoinMap software for uniformity with the purpose of integrating all three maps into a single consensus map. The chi-square cutoff used for this population was also 9.2 and all the markers showed the expected segregation ratio. The grouping of chromosomes was done on the basis of a recombination frequency of 0.35 and markers that did not conform to the stable order were discarded.

The three individual genetic maps were then merged to generate the consensus linkage map using MergeMap online [19] by converting the individual maps into directed acrylic graphs (DAGs) that were then merged into a consensus graph on the basis of their share vertices. While constructing the consensus linkage map, “weights” were assigned to the three populations based on the level of confidence we had on their marker orders. Hence, population G was given a weight of 5.0, followed by population A with a weight of 3.0, and population R with a weight of 1.0. The final map was drawn using software MapChart [20].

### Comparison of consensus linkage groups with published genome pseudomolecules of pigeonpea

A comparative analysis was carried out using sequence information from the markers included in the present consensus linkage map with published pigeonpea genome pseudomolecules of the International Crops Research Institute for the Semi-Arid Tropics (ICRISAT) [7]. Mapped SNP flanking sequences from the 1,536-plex and 768–plex Illumina GoldenGate assays, primer sequences of SSRs, and flanking sequences of 319 RAD-SNP markers were used for comparative analysis. The sequences of all 932 markers in the consensus map were searched for in the ICRISAT chromosome pseudomolecules using BLASTn with earlier published [21] pre-optimized parameters (-v 1, -b 1, -E 1, -q 1, -r 1, -F, F–e, and 1e-10). A chromosome wise list of our (NRCPB) markers matched in the ICRISAT pseudomolecules was also prepared.

## Results

The main objective of this study was to construct a high-density SNP linkage map of pigeonpea for which we first constructed two new component linkage maps using two different mapping populations, (i) Asha/UPAS 120 (Population A) and (ii) Pusa Dwarf/H2001-4 (Population R). The linkage map of population A comprised 725 markers and that of population R comprised 136 markers. These two new maps were then integrated with a third population, a redrawn map of Pusa Dwarf/HDM04-1 population (Population G) [11], to develop a high-density consensus linkage map of the 11 chromosomes of pigeonpea.

### Linkage map of Asha/UPAS 120

Genotyping data were generated from 92 F_2_ individuals of population A (Asha/UPAS 120) to identify 10,786 marker loci using different marker systems including GoldenGate genic SNP, RAD-GBS SNP, ASSR and HASSR markers (Table 1). From the 768-plex GoldenGate assay, 159 polymorphic markers were analyzed, of which 106 were included in the linkage map, whereas from the 1,536-plex GoldenGate assay, 382 markers were found to be polymorphic, of which 197 were included in the linkage map. Overall, out of the 2,304 SNP loci from the two GoldenGate assays 541 loci (23.48%) were polymorphic between parental varieties Asha and UPAS 120. We were expecting higher polymorphism because SNPs in the GoldenGate assay were identified by an in-silico comparison of Asha and UPAS 120 transcript sequences; however, the lower than expected success may be attributed to heterogeneous sample of variety Asha that was used in the transcript sequencing. For GBS-RAD based genotyping 26.5 Gb and 21.3 Gb high quality reads were generated with the *Apek*I and *Pst*I enzymes, respectively. In total 7,500 SNPs were identified, of which only 1,207 SNPs could be used for map making, the remaining markers were discarded owing to segregation distortion and a high number of missing data points due to lack of commonly genotyped markers across the 92 individuals in the mapping population A. Finally, only 319 GBS–RAD SNPs were included in the map of population A (S1 Table). Of the 71 ASSR and 151 HASSR markers screened, only 65 (29%) were found to be polymorphic and 41 were included in the linkage map. The number of mapped markers in the 11 different linkage groups (LGs) ranged from 24 in LG3 to 124 in LG1. The minimum map length was observed in LG3 (62.0 cM) whereas the maximum map length was observed in LG11 (127.2 cM). The largest interval was observed in LG9 (13.2 cM) and the shortest interval was observed in LG08 (4.3 cM). In total, 725 markers were included in the map with population A, which covered a map length of 1,102.2 cM and an adjusted map length of 1,105.5 cM with an average map interval of 1.39 cM (Table 1; S1 Fig). This provides a new dense genetic map of pigeonpea based on a single intraspecific mapping population.

**Table 1:** Features of the component genetic maps along with their contribution of markers to the consensus pigeonpea genetic map.

Number of markers that contributed to the component genetic map (Number of markers used in the consensus map)				Consensus Map	
Linkage Group	Asha/ UPAS 120 (population A)	Pusa Dwarf/ H2001-4 (population R)	Pusa Dwarf/ HDM04-1 (population G)	Number of Markers	Map Distance (cM)
1	94(60)	22(-)	56(29)	127	136.9
2	124(94)	22(-)	35(16)	144	191.6
3	24(17)	7(-)	32(24)	52	115.03
4	65(53)	13(-)	27(21)	89	124.76
5	58(50)	10(-)	21(17)	78	102.23
6	58(45)	8(-)	20(12)	71	117.361
7	81(68)	6(-)	22(11)	94	119.02
8	81(35)	8(1)	23(11)	63	104.99
9	43(32)	11(1)	18(12)	58	135.41
10	53(38)	15(-)	16(12)	67	156.48
11	77(61)	14(1)	21(9)	89	140.82
**Total**	**725(553)**	**136(3)**	**291(174)**	**932**	**1411.83**

### Linkage map of Pusa Dwarf/H2001-4

The population R (Pusa Dwarf/H2001-4) included 94 F_2_ individuals that were genotyped using only the 1,536-plex GoldenGate SNP assay, and 186 polymorphic loci (12.1%) were found, owing to the narrow genetic base of pigeonpea varieties. Only 136 of these loci were included in the linkage map, with the remaining 50 markers discarded owing to higher missing values in the genotyping data and segregation distortion. The number of mapped markers in the 11 different linkage groups ranged from 6 in LG7 to 22 in LG1 and LG2. The minimum map length was observed in LG7 (39.66 cM), whereas the maximum map length was observed in LG2 (174.1 cM). The largest interval was observed in LG3 (34.80 cM) and the shortest interval was observed in LG01 (10.7 cM). The total map length for population R was 929.82 cM with an adjusted map length of 943.58 cM and average marker interval of 8.05 cM (S2 Fig). This is also a new intraspecific molecular linkage map of pigeonpea with 136 SNP markers.

### Linkage map of Pusa Dwarf/HDM04-1

The linkage map of population G (Pusa Dwarf/HDM04-1) with 296 markers was previously drawn using MapDisto software and has been used for the mapping of QTLs for plant height, stem branching, and maturity time in pigeonpea [11]. The map was redrawn in this study using JoinMap software to preserve uniformity when integrating the three maps into a single consensus map. Seven of the 296 markers were discarded from the earlier published map and two new markers were added, providing a total number of 291 markers. The seven markers that were removed in the current map were ASNP1310 from LG4; ASNP1818, ASNP234, ASNP242, and ASSR109 from LG5; ASNP280 from LG8; and ASSR81 from LG1. These markers were removed because they did not conform to the most stable marker order. Excluding these markers and including the two additional ones provided the most stable marker order for the individual map of population G using JoinMap.

The order of the markers was similar to the previously published map but the total map distance was reduced from 1,406.7 cM to 923.9 cM (S3 Fig). The map length was also reduced because JoinMap uses a different algorithm than MapDisto and the current linkage distances were slightly smaller than those in the previously published map. The number of mapped markers in the 11 different linkage groups ranged from 16 in LG11 to 56 in LG1 (Table 1). The minimum map length was observed in LG11 (52.2 cM) and maximum map length was observed in LG1 (196.6 cM). The largest gap size was observed in LG10 (25.4 cM) and the smallest one was observed in LG6 (15.1 cM). The reconstructed map has a total map length of 923.9 cM and adjusted map length of 930.3 cM, with average marker interval of 2.93 cM (S3 Fig).

### Consensus linkage map

**Fig 1:**
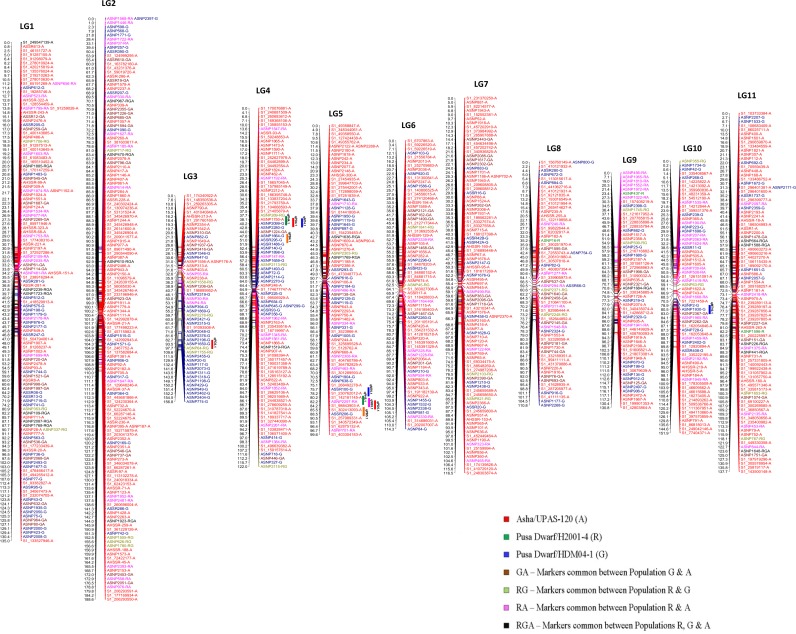
The consensus linkage map of 932 markers that are both common and unique to the three pigeonpea mapping populations. QTLs for plant height (PH), maturity time (MT), number of pods (PD), number of primary branches (PB), and number of secondary branches (SB) identified by Giriraj et al. [11] are indicated by different colored bars. Green, blue, red, orange, and magenta colored bars represent the QTLs for PH, MT, PD, PB, and SB, respectively.

Three individual genetic maps of populations A, R, and G were merged to generate an intraspecific consensus map using MergeMap software by converting the individual population maps into DAGs that were then merged into a consensus graph on the basis of their shared vertices. In total, 1,152 markers (1,083 SNP and 69 SSR) were included in the three linkage maps, of which 158 conflicting markers were discarded during the process of merging the individual maps using MergeMap. The consensus linkage map has 932 markers that included 863 SNP and 69 SSR loci **(**Table 1; Fig 1**)**. In the consensus map a total of 202 (21.6%) markers were common between the populations, out of which 69 markers were common between population G and A, 87 were common between populations R and A, 27 were common between populations R and G, and 19 markers were common to all three populations; leaving the remaining 730 (78.4%) that were unique to the individual mapping populations themselves.

To avoid confusion with multiple nomenclatures, we have retained the original numbering of linkage groups LG1 to LG11 from our previously published map with population G [11]. The total number of markers in the individual linkage groups in the consensus map ranged from 52 in LG3 to 144 in LG2. LG2 exhibited the maximum adjusted map length of 191.60 cM, whereas LG5 with 78 markers showed the minimum map length of 102.23 cM. The average marker interval ranged from 1.07 cM for LG1 to 2.3 cM for LG9 and LG10. Non-uniform distribution of markers was evident in all of the linkage groups. The largest map interval was 27.42 cM in LG1 followed by 13.25 cM in LG9; however, two are more markers were collocated at 17 different positions in the consensus map (S2 Table). The detailed statistics for each linkage group in the consensus linkage maps along with number of markers contributed by each component genetic map is shown in Table 2.

**Table 2:** Chromosome-wide details of a predominantly SNP based intraspecific consensus linkage map of pigeonpea constructed using three different F_2_ populations: Asha/UPAS 120, Pusa Dwarf/H2001-4, and Pusa Dwarf/HDM04-1.

Linkage group	No. of markers	Map length (cM)	Adjusted map length (cM)	Average map interval (cM)	Largest map interval (cM)
LG1	127	135.01	136.90	1.07	27.42
LG2	144	188.57	191.60	1.33	6.71
LG3	52	110.69	115.03	2.21	8.09
LG4	89	121.99	124.76	1.40	6.97
LG5	78	99.64	102.23	1.31	6.93
LG6	71	114.34	117.61	1.65	6.14
LG7	94	116.51	119.02	1.26	9.17
LG8	63	101.71	104.99	1.66	4.24
LG9	58	130.82	135.41	2.33	13.25
LG10	67	151.88	156.48	2.33	9.05
LG11	89	137.69	140.82	1.58	7.06
**Total**	**932**	**1,408.8**	**1,411.83**	**1.51**	**27.42**

The consensus linkage map covered a total genome map length of 1,408.85 cM, which converted to an adjusted map length of 1,411.83 cM using method 4 from Chakravarti et al.[22]. The average marker interval was 1.51 cM, which was very useful for anchoring the genome and genetic studies in pigeonpea. From earlier published QTLs in pigeonpea by Giriraj et al (thirteen)[11], Bohra et al (four) [10] and Gnanesha et al (six) [23], we could easily place 11 out of 13 QTLs reported by Giriraj et al. [11] for plant height (PH), maturity time (MT), number of pods (PD), number of primary branches (PB), and number of secondary branches (SB) in our consensus map. We could not place other QTLs reported by Bohra et al. [10] and Gnanesh et al. [23], due to the lack of common markers/marker sequence information (Fig 1).

**Fig 2:**
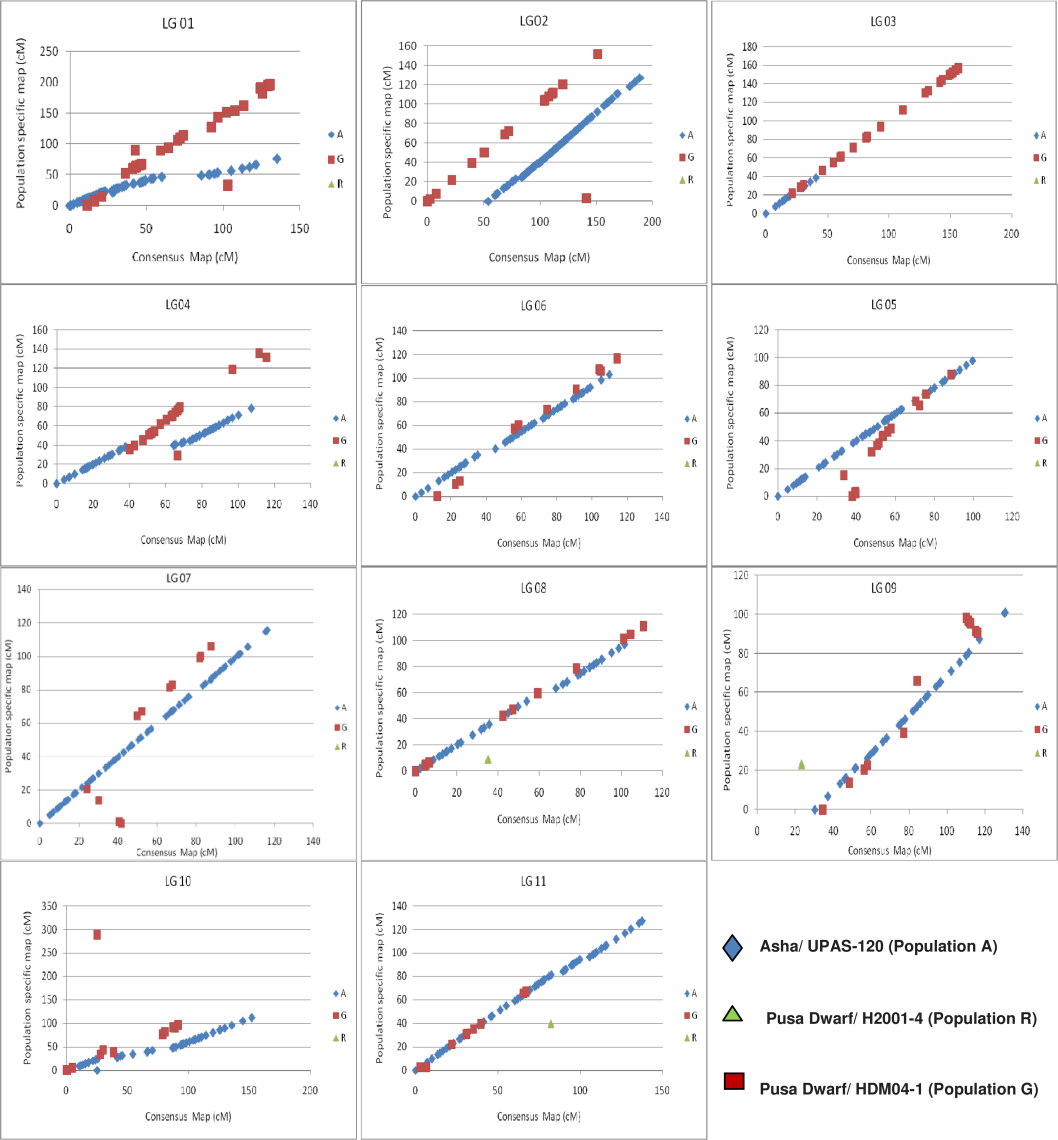
Graphical representation of correspondence between component maps and consensus map. Scatter plot showing the extent of the correlations between the consensus genetic map and component genetic maps. The markers from component genetic maps of the populations A, G, and R are shown as blue diamonds, red squares, and green triangles, respectively.

A detailed comparison of all linkage groups across all the maps is presented in Fig 2. A high degree of correlation was observed for most of the LGs between consensus and component LGs. A high degree of correlation was observed for all the LGs and showed a good agreement for both marker orders and marker positions between consensus and component LGs.

**Fig 3:**
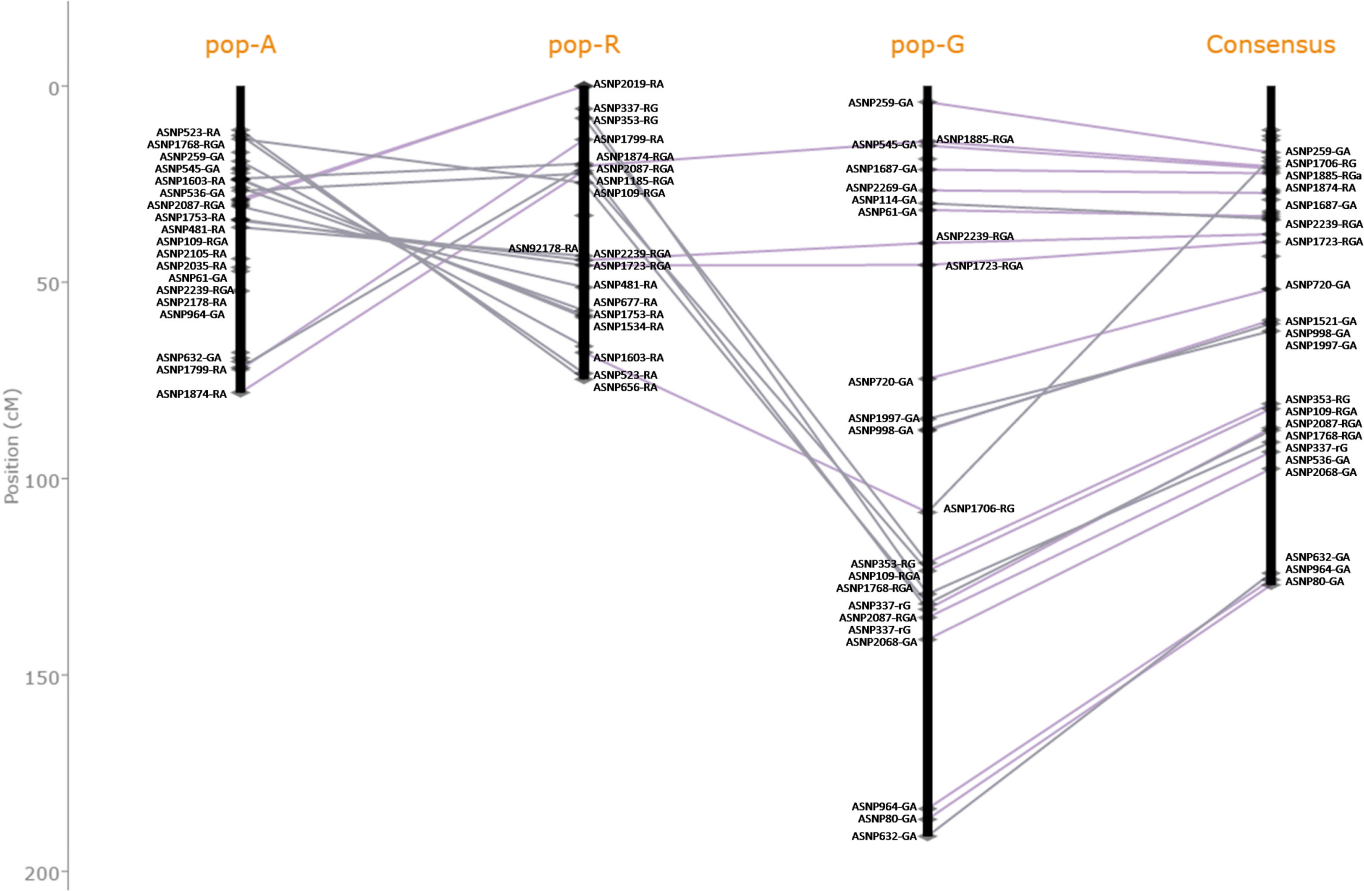
Correspondence among consensus and component maps of LG1. Depiction of common markers between the consensus and individual genetic maps for the LG01 pigeonpea linkage group aligned together using the Genetic Map Comparator software.

The consistency of marker order and possible rearrangement between the component maps and consensus genetic map was compared using the Genetic Map Comparator software [24]. Between the three maps, a total of 19 markers were common to all the maps and 183 markers were common in pairs of maps. For example, the correspondence among consensus and component maps of LG01 is shown in Fig 3. The component maps for the remaining linkage groups is shown in S4 Fig.

### Correspondence between NRCPB and ICRISAT linkage groups in pigeonpea

Prior to the Indo-US AKI Pigeonpea Genomics Initiative (PGI) there was no published molecular linkage map of pigeonpea [25]. However, after the AKI-PGI project commenced, two different groups at the National Research Centre on Plant Biotechnology (NRCPB) in New Delhi, India, and the International Crop Research Institute for Semi-Arid Tropics (ICRISAT) in Hyderabad, India, published independent pigeonpea molecular linkage maps; hence, it was necessary to examine the consistency between the NRCPB and ICRISAT linkage maps. The numbering used for linkage groups in the consensus linkage map described here is consistent with the published NRCPB linkage map [11]. The nucleotide sequences of 932 markers in the present map were compared using a BLAST search of the ICRISAT pigeonpea genome pseudomolecules CcLG1-CcLG11. Clear identities were obtained for only 391 of the markers, indicating that the ICRISAT pseudomolecules cover only approximately 41% of the pigeonpea genome when considering random genomic distribution of the NRCPB markers. Furthermore, each of the NRCPB linkage groups matched with an ICRISAT linkage group, but with different numbering except for NRCPB LG2, which had identical numbering as that in the two maps (Table 3). Interestingly, ICRISAT CcLG2, in addition to having similarity to the NRCPB LG2, also matched with NRCPB LG6. This suggests that either NRCPB LG2 and LG6 were falsely separated into two different chromosomes, or more likely, because of the very tightly linked markers in the NRCPB genetic map, the ICRISAT pseudomolecules for CcLG2 may in fact be a fusion of two distinct chromosomes. To resolve this potential inaccuracy, we took five markers from each of the terminal ends of NRCPB LG2 and NRCPB LG6, reanalyzed using JoinMap that have shown that these markers belong to two different groups and that the ICRISAT CcLG2 was probably a false fusion of the two linkage groups. There was a large number of cross-matching of markers from a single NRCPB linkage group with multiple pseudomolecules of ICRISAT, suggesting poor assembly of the pseudomolecules. This needs further assessment to generate a high-quality reference genome for pigenopea.

**Table 3:** Correspondence between NRCPB pigeonpea linkage groups and ICRISAT chromosome pseudomolecules (CcLG1-11).

NRCPB linkage map			ICRISAT Pseudomolecule	
Linkage Group	No. of markers	No. (%) of markers matching in ICRISAT pseudomolecules	Linkage group (no. of matched markers)	Size of the best matched LG (Mb)
LG1	127	49 (38)	**LG11(44),** LG02(02), LG07(02), LG06(01)	48
LG2	144	77(53)	**LG02(51),** LG03(11), LG07(04), LG09(03) LG11(03), LG06(02), LG10(02), LG01(01)	36
LG3	52	23(44)	**LG08(18),** LG02(03), LG09(01), LG06(01)	20
LG4	89	40(44)	**LG03(26),** LG11(09), LG08(02), LG10(02), LG02(01)	29
LG5	78	29(37)	**LG07(13),** LG11(06), LG03(03), LG02(03), LG01(02), LG08(01)	19
LG6	71	16(22)	**LG02(11),** LG09(02), LG01(02), LG11(01)	36
LG7	94	25(26)	LG11(05), LG08(05), LG01(04), LG09(04), LG05(03), LG10(02), LG02(01), LG06(01)	-
LG8	63	32(50)	**LG06(27)**, LG11(20), LG10(02), LG07(01)	23
LG9	58	26(44)	**LG10(10),** LG11(03), LG01(02), LG02(01), LG03(01), LG08(01)	22
LG10	67	36(53)	**LG04(24),** LG06(04), LG03(03), LG11(02), LG10(02)	12
LG11	89	38(42)	**LG01(28),** LG10(05), LG11(02), LG08(02), LG02(01)	17
**Total**	**932**	**391 (41%)**	** **	** **

## Discussion

Availability of genomic resources including high-resolution genetic and physical maps is essential for accelerating the breeding of improved crop varieties. In this study, we report an intraspecific consensus linkage map based on three different F_2_ mapping populations (A, R, and G) of pigeonpea that surpasses the previously published genetic maps with a significantly higher number (932) of markers, including a large number of highly reproducible SNP markers covering a total map length of 1,411.83 cM. We expected a high proportion of polymorphic loci in population A because the SNPs in the assays were identified after in silico comparison of the parental varieties Asha and UPAS 120 transcriptome sequences. Although the observed 20.87% polymorphism rate in the GoldenGate assay for population A was less than expected due to heterogeneous samples used in the generation of transcriptome data, it was still significantly higher than the populations R and G, where in-silico polymorphism searched was not feasible.

Unfortunately, a majority of RAD-GBS markers were discarded owing to a high number of missing data in the progeny. A low level of useful polymorphism was observed with the RAD-GBS sequencing method as compared to the GoldenGate genotyping method, mainly because it was difficult to find a large number of common markers in the population genotyped by RAD sequencing.

The linkage map of population G has already been published using MapDisto software. Hence, we re-constructed this map using JoinMap for uniformity between component maps and found that the loci order and their map positions were quite similar to that of the published map, with the exception of the exclusion of seven markers and inclusion of two additional markers. These differences resulted in a total of 291 markers in our new component map of population G, instead of 296 markers in the previous map.

The success of building a dense consensus linkage map by merging multiple individual genetic maps depends on the availability of common markers between the populations for each of the linkage groups. For this study the nomenclature of markers should be the same and their position should be similar across all populations [26]. We followed the convention of at least three common markers in each linkage group and then proceeded to make the final map using MergeMap in a single run. It was observed that a consensus order was not possible unless the markers with conflicting positions between maps were removed. Hence, the marker order was filtered and rearranged until the most agreeable order was established, incorporating the maximum possible number of retained markers. In this way, a consensus linkage order among markers was achieved for 932 markers and the remaining 158 markers were discarded owing to their conflicting locations between maps. The total adjusted length of the consensus genetic map was 1,411.83 cM covered by the 11 linkage groups. This new consensus map is now has the highest marker density among the published linkage maps of pigeonpea.

The 932 marker sequences included in the present map were compared to the ICRISAT pigeonpea pseudomolecules CcLG1-CcLG11; however, we observed significant matches with only 391 of the markers, indicating that the ICRISAT pseudomolecules cover only approximately 41% of the pigeonpea genome, considering that there is a random genomic distribution of markers in the NRCPB map. There was one-to-one correspondence in nine of the eleven linkage groups between NRCPB and ICRISAT maps, although the numbering of seven linkage groups was different from that of the ICRISAT linkage groups. There were large numbers of cross matches of markers from a single NRCPB linkage group with multiple ICRISAT pseudomolecules, suggesting that the poor assembly of the pseudomolecules needs further attention to generate a high-quality reference genome for pigeonpea that can be used for genetic and breeding applications.

Our results suggest that the LG2 pseudomolecule is the fusion of two different linkage groups that correspond to the NRCPB linkage groups two and six, which may have been joined together due to a misassembled scaffold. When we analyzed the markers at the junction of NRCPB LG2 and LG6 homology, on the ICRISAT LG2 we found that these markers belong to different linkage groups, as they do not show any linkage to each other. The NRCPB LG7 did not clearly matched with any ICRISAT linkage group; but instead it showed matches with several ICRISAT chromosomes.

In summary, a largely SNP-based, high-density, intraspecific consensus linkage map of the pigeonpea genome was constructed, which included 932 loci with an average marker interval of 1.51 cM. This is the first map of pigeonpea using RAD-SNP markers and it will be helpful in genetic mapping and breeding applications for this species and for anchoring the reference genome of pigeonpea. Furthermore, the GBS-RAD SNP approach was found to be tedious and the least productive because a large proportion of SNPs was excluded from the map due to a lack of genotyping data owing to low fold sequence coverage. The present map is the first high-density, intraspecific linkage map of pigeonpea that is based on a large number of genic-SSR and SNP markers that cover a high genome length of 1,411 cM. Our results also emphasize the need for utilizing SNP markers for the creation of ultra high-density linkage maps of pigeonpea because of the poor polymorphism of SSR markers. With the availability of a high-density consensus linkage map of pigeonpea, molecular breeding applications for pigeonpea will be improved by the use of DNA markers linked to the traits of agronomic importance.

## Supporting information

S1 Table Sequence information of the TASSEL data used in population A. (XLS)

S2 Table Markers collocated at 17 positions in the consensus map. (XLS)

S1 Fig Details of the component genetic map of Pop A. (TIF)

S2 Fig Details of the component genetic map of Pop R. (TIF)

S3 Fig Details of the component genetic map of Pop G. (TIF)

S4 Fig Correspondence among consensus and component maps of Linkage Groups LG02 to LG11. (PDF)
