# Supplementary figures and images for: A high-density intraspecific SNP linkage map of pigeonpea (Cajanas cajan L. Millsp.)
Source: PLoS One. 2017 Jun 27;12(6):e0179747. doi: 10.1371/journal.pone.0179747 (PMC5487049; doi:10.1371/journal.pone.0179747)

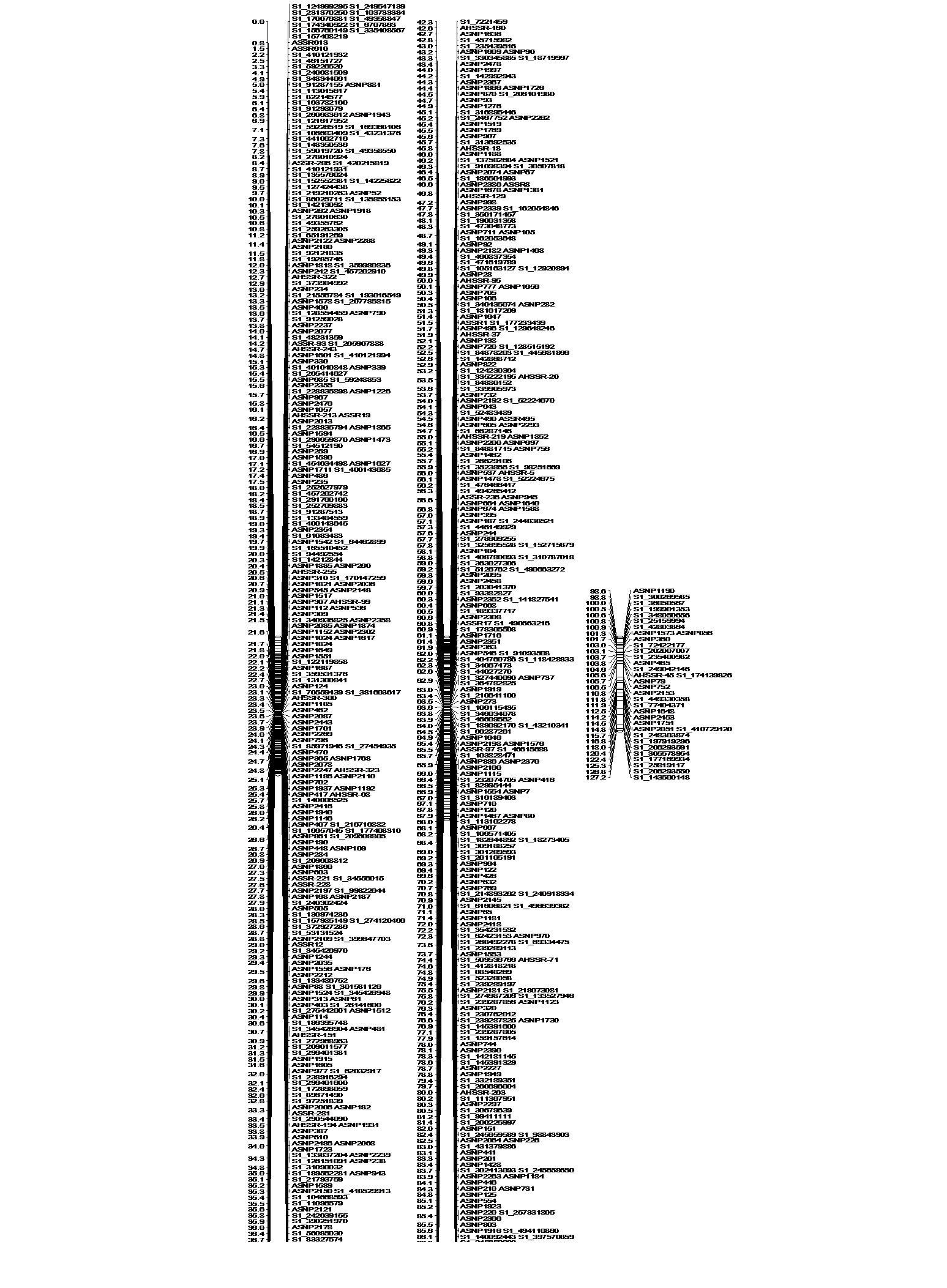

Supplement: S1 Fig — (TIF) [file pone.0179747.s003.tif]

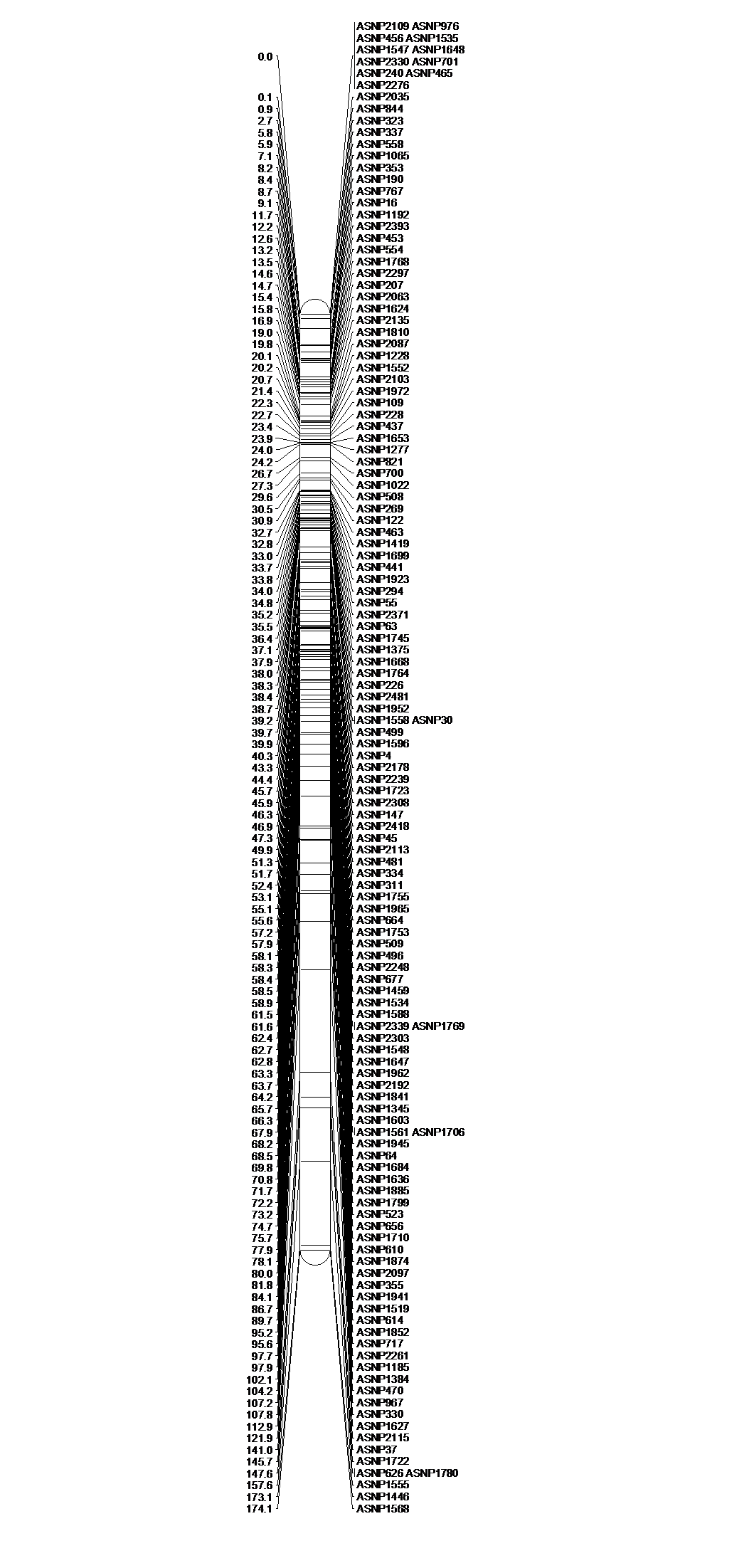

Supplement: S2 Fig — (TIF) [file pone.0179747.s004.tif]

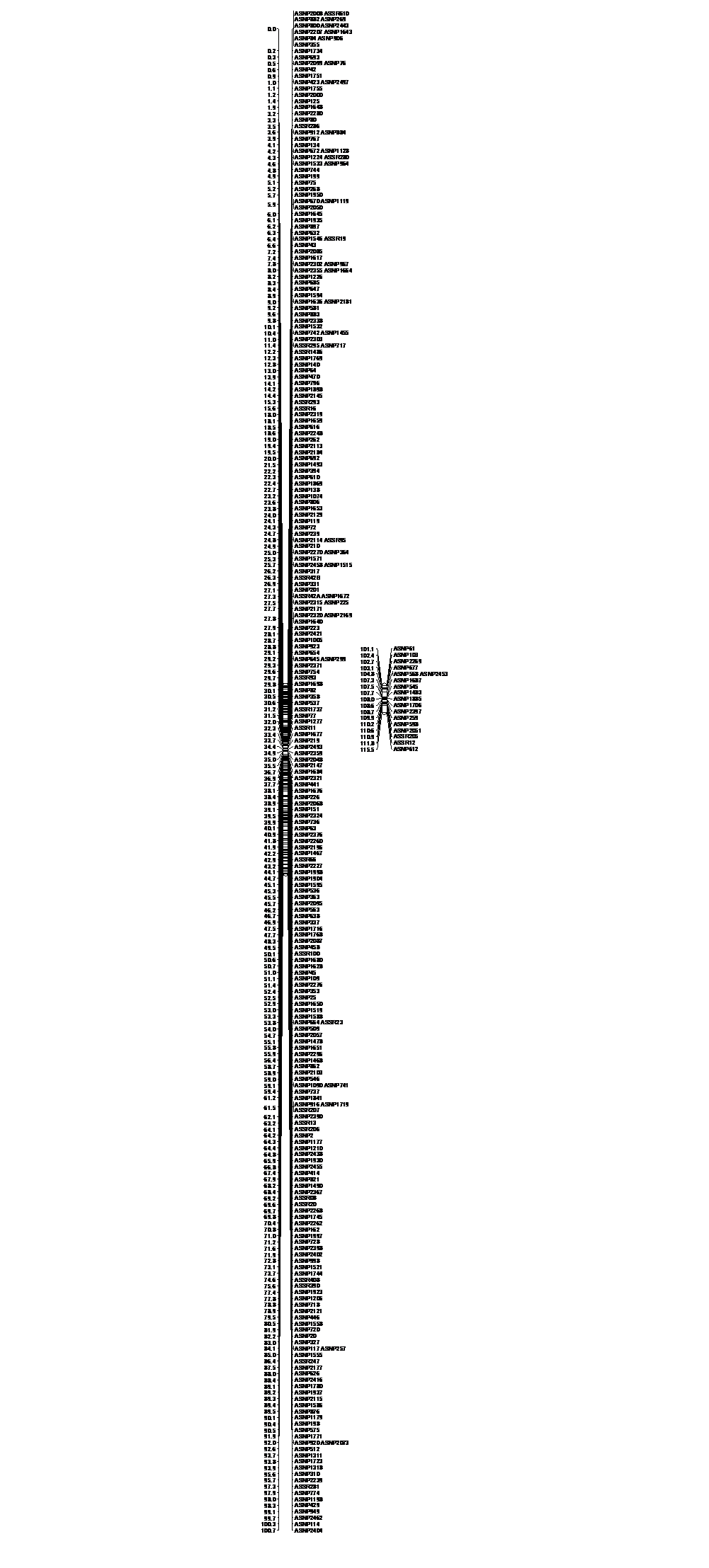

Supplement: S3 Fig — (TIF) [file pone.0179747.s005.tif]

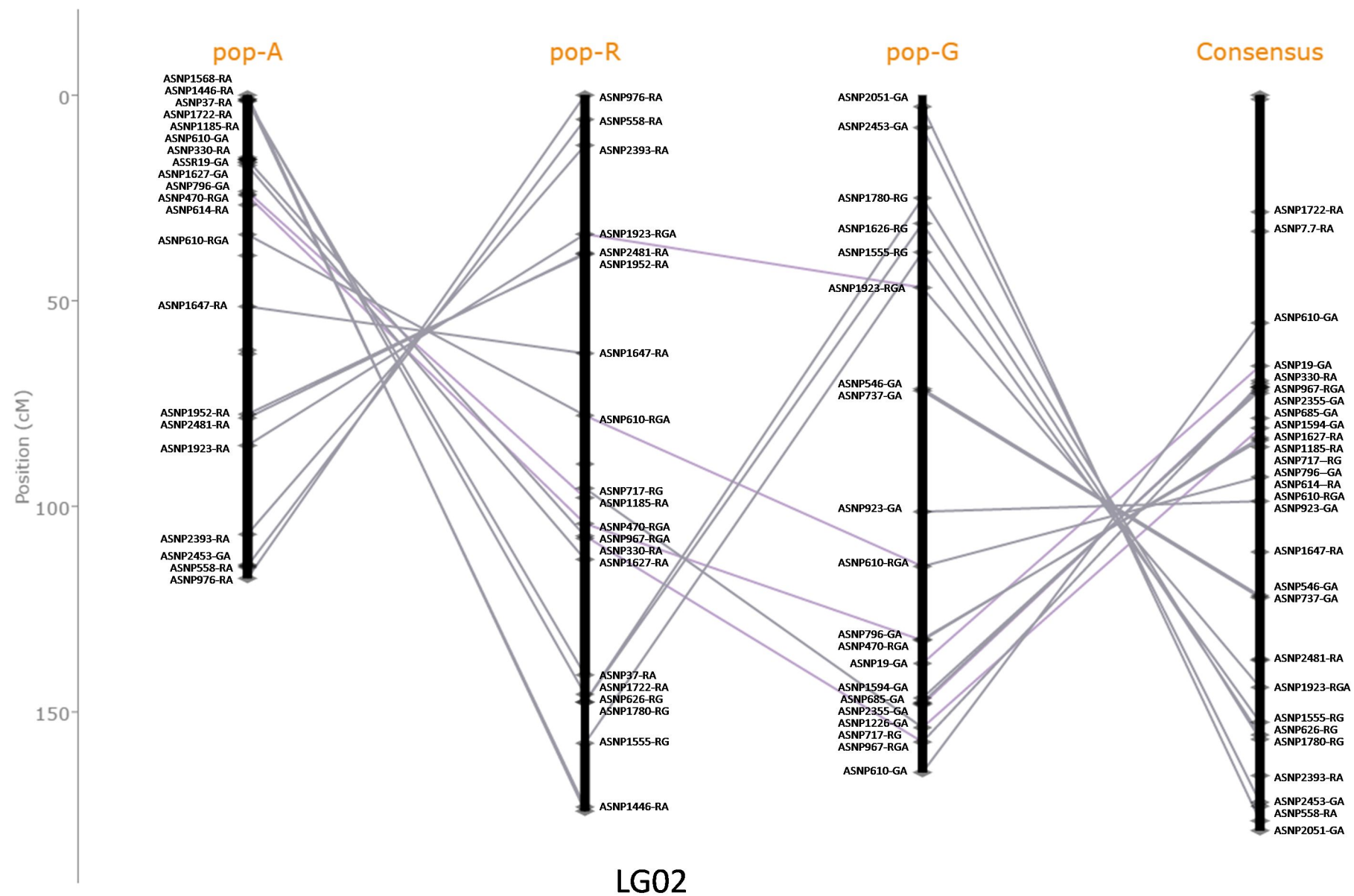

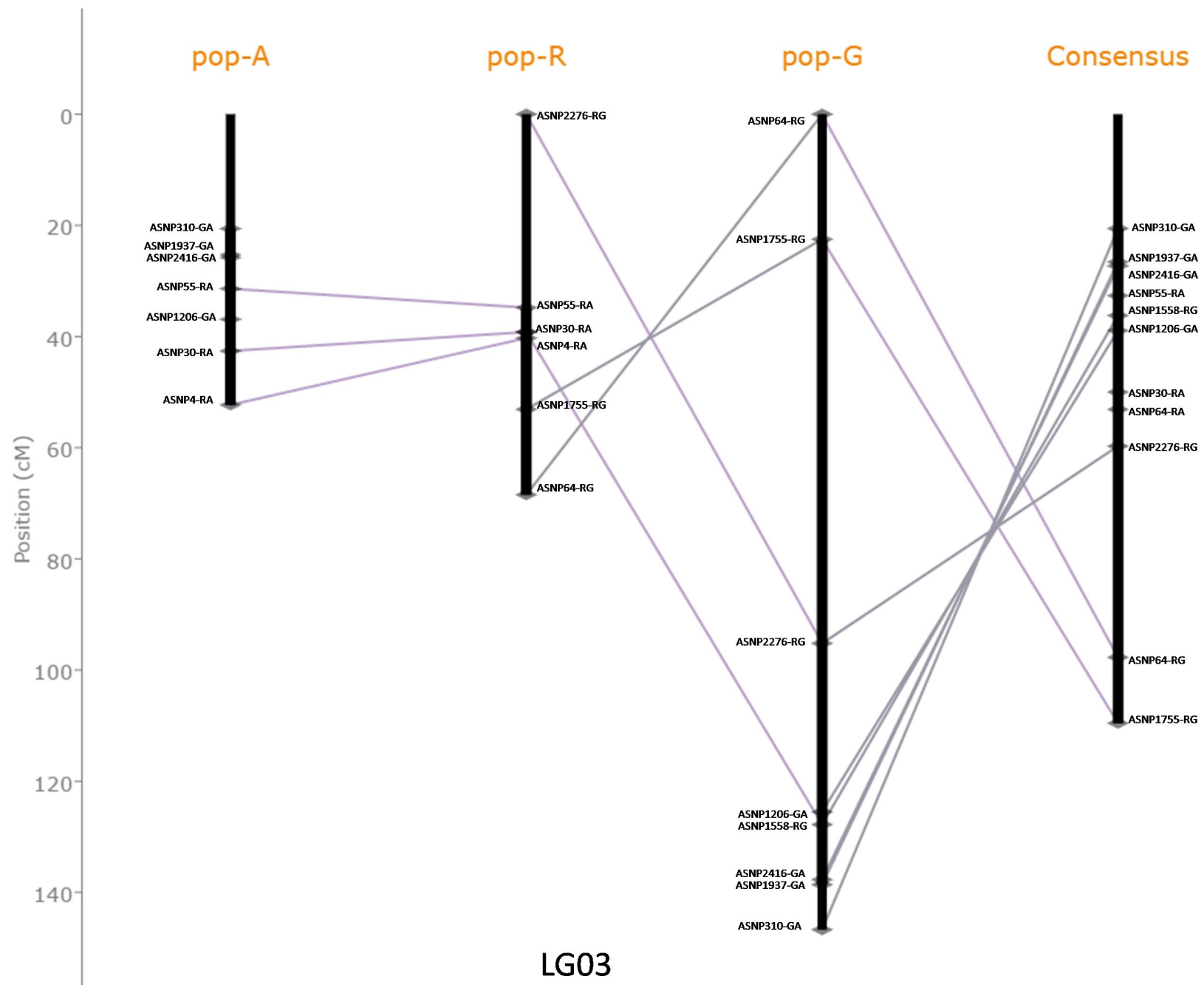

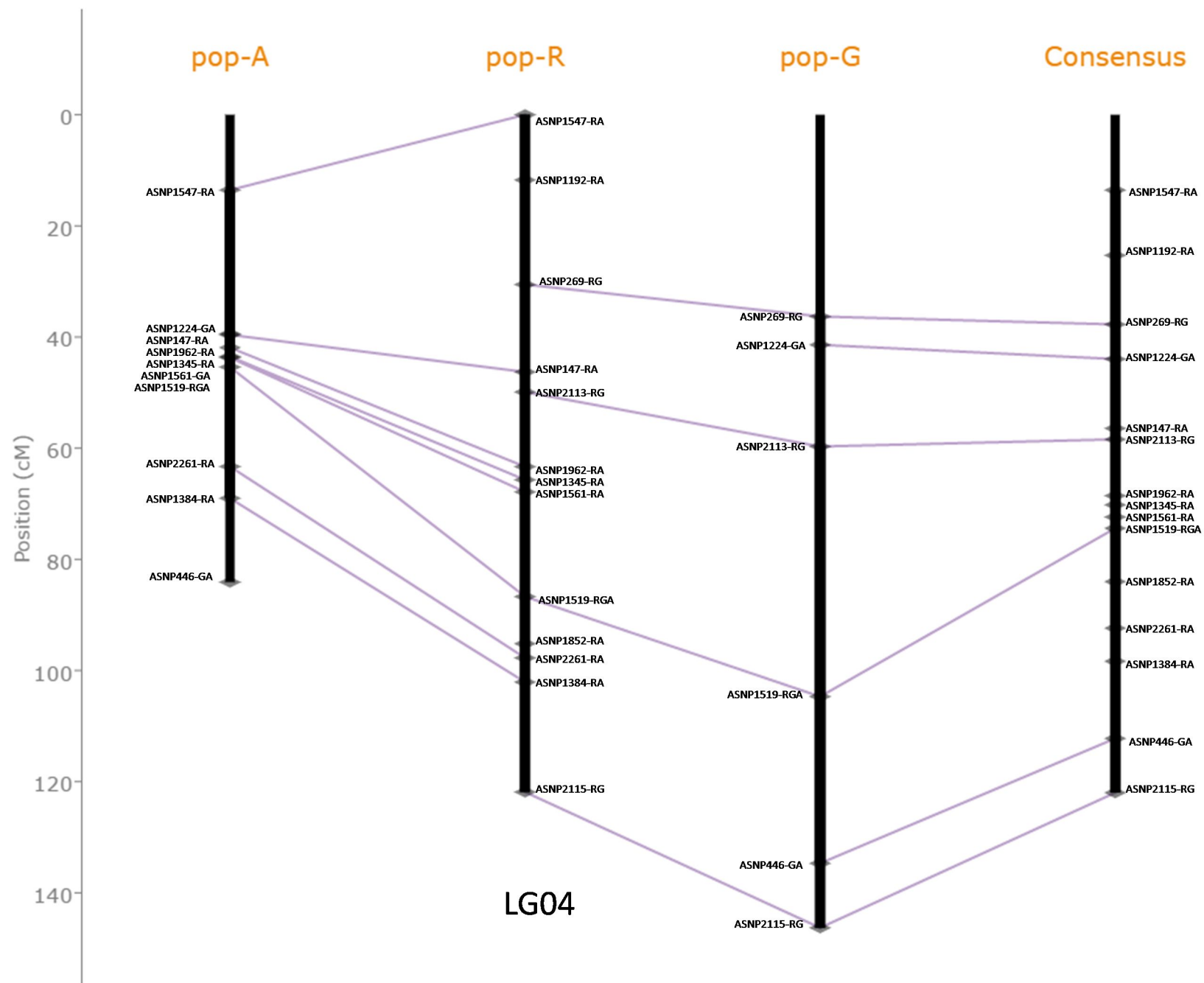

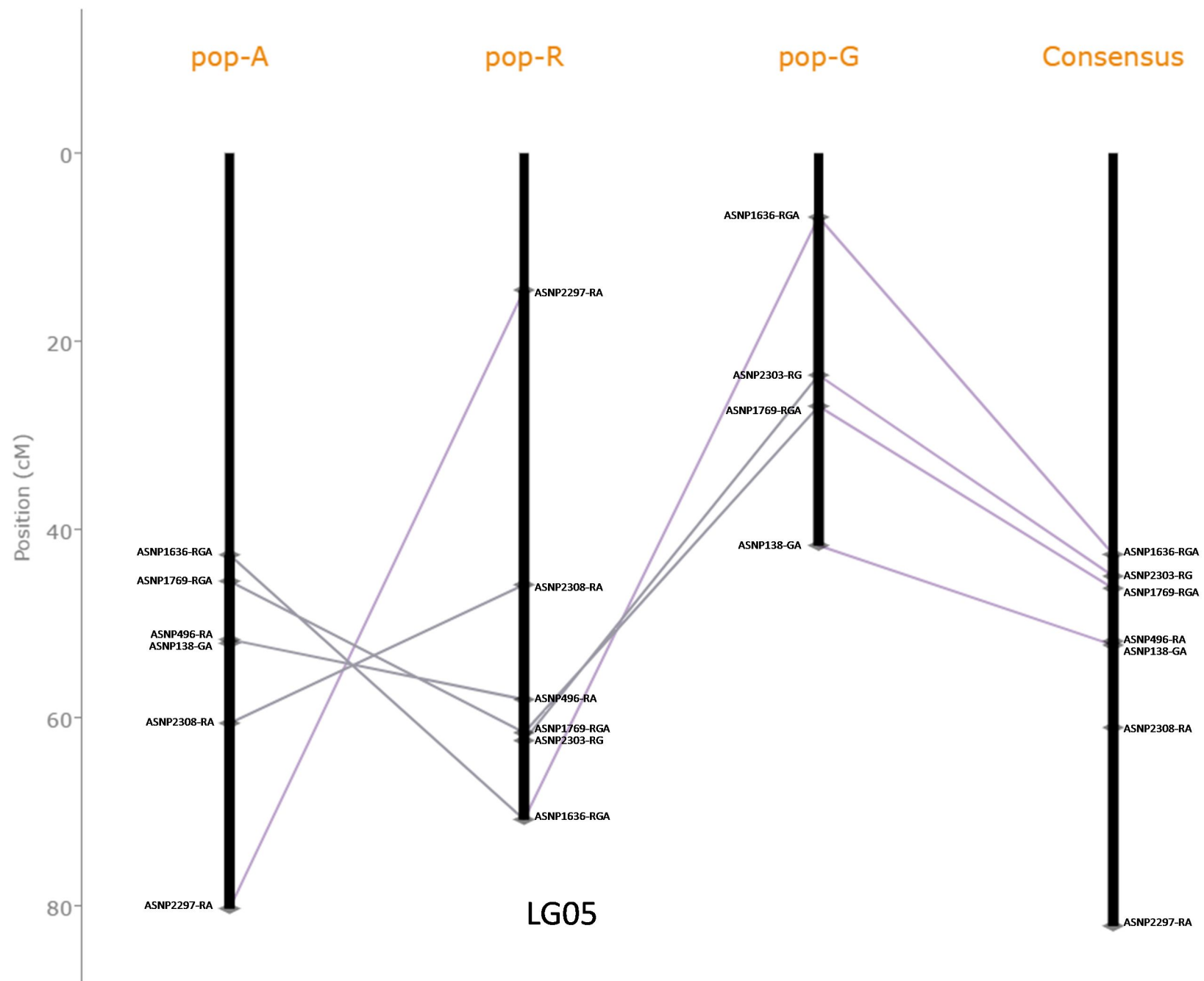

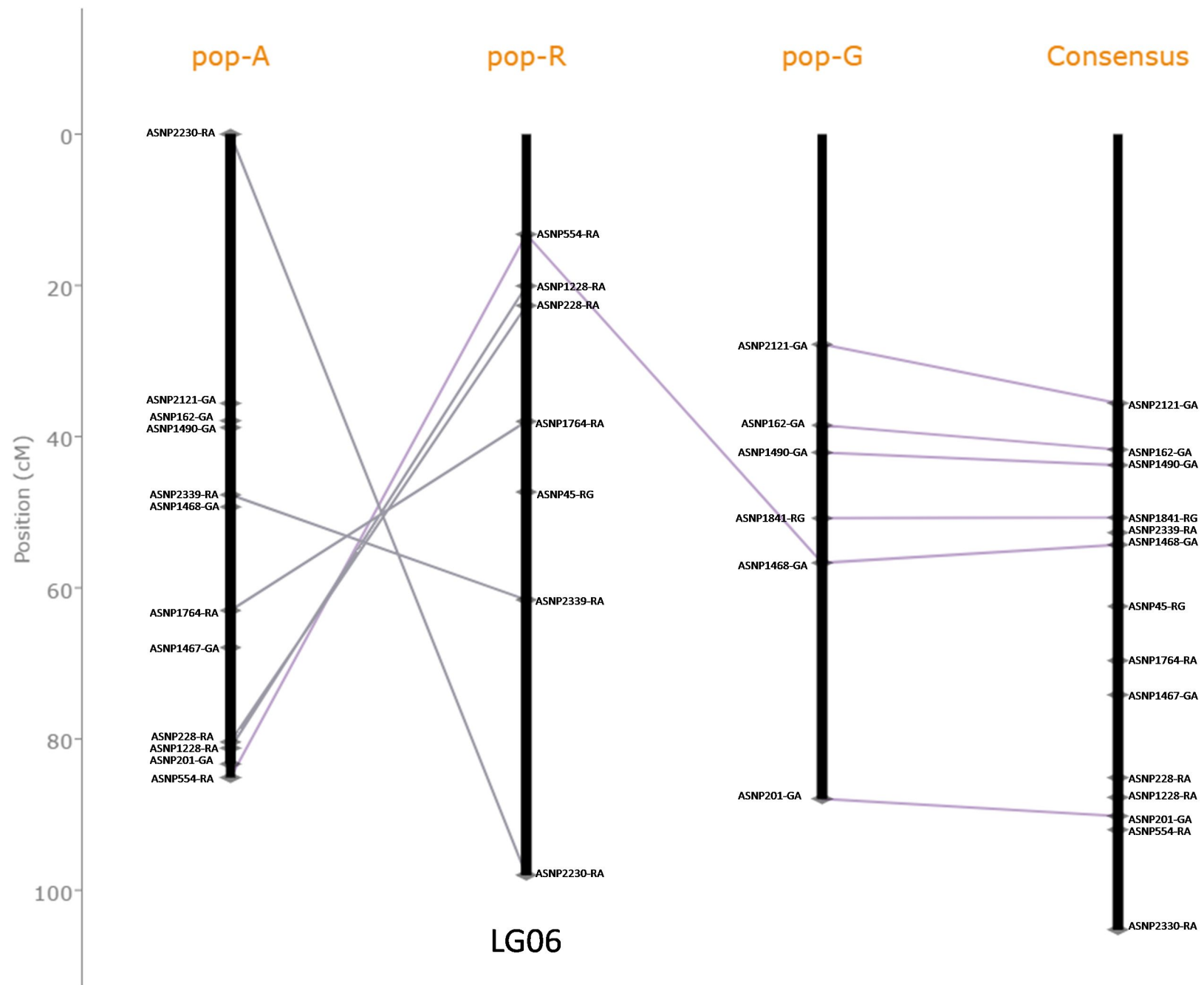

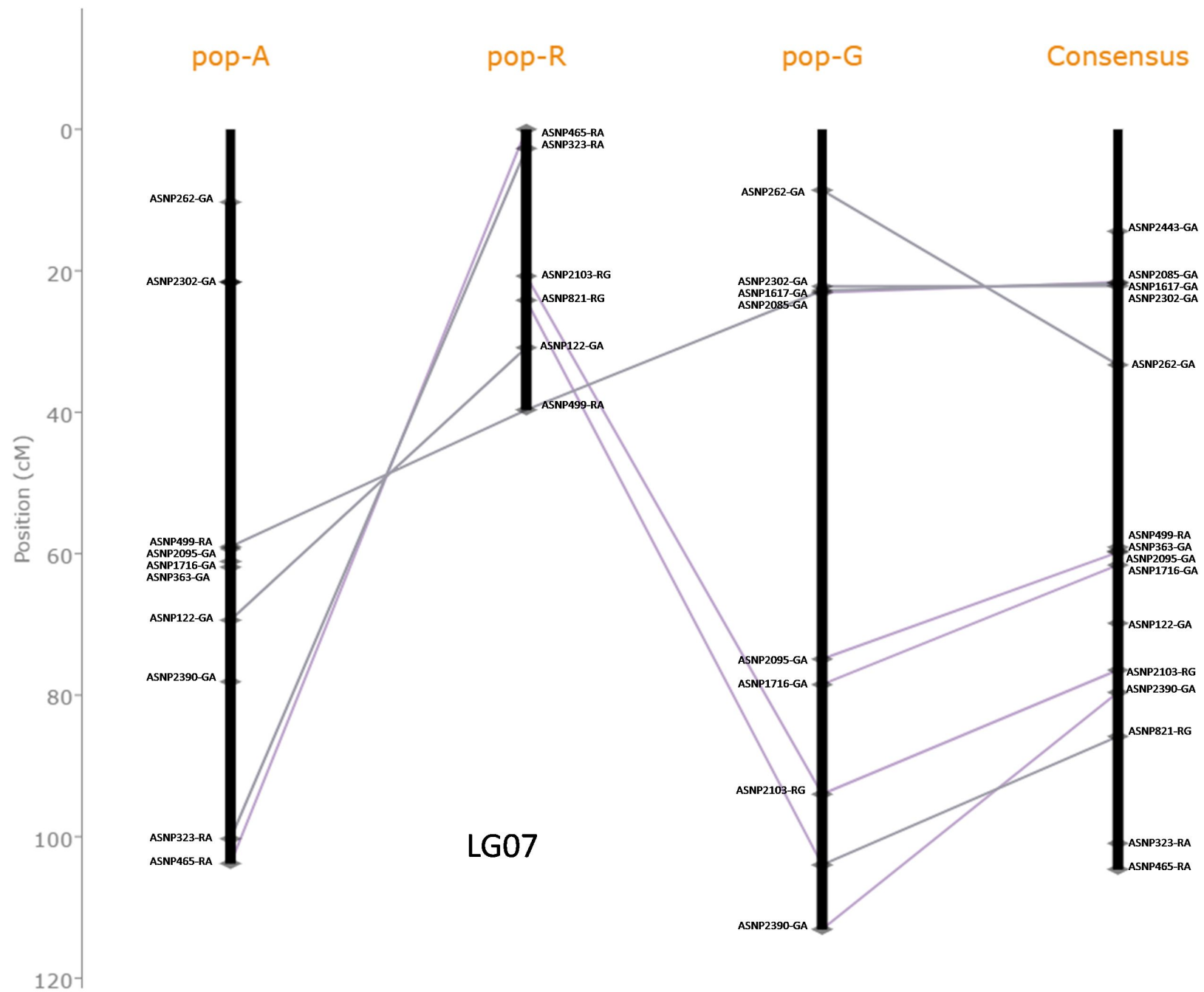

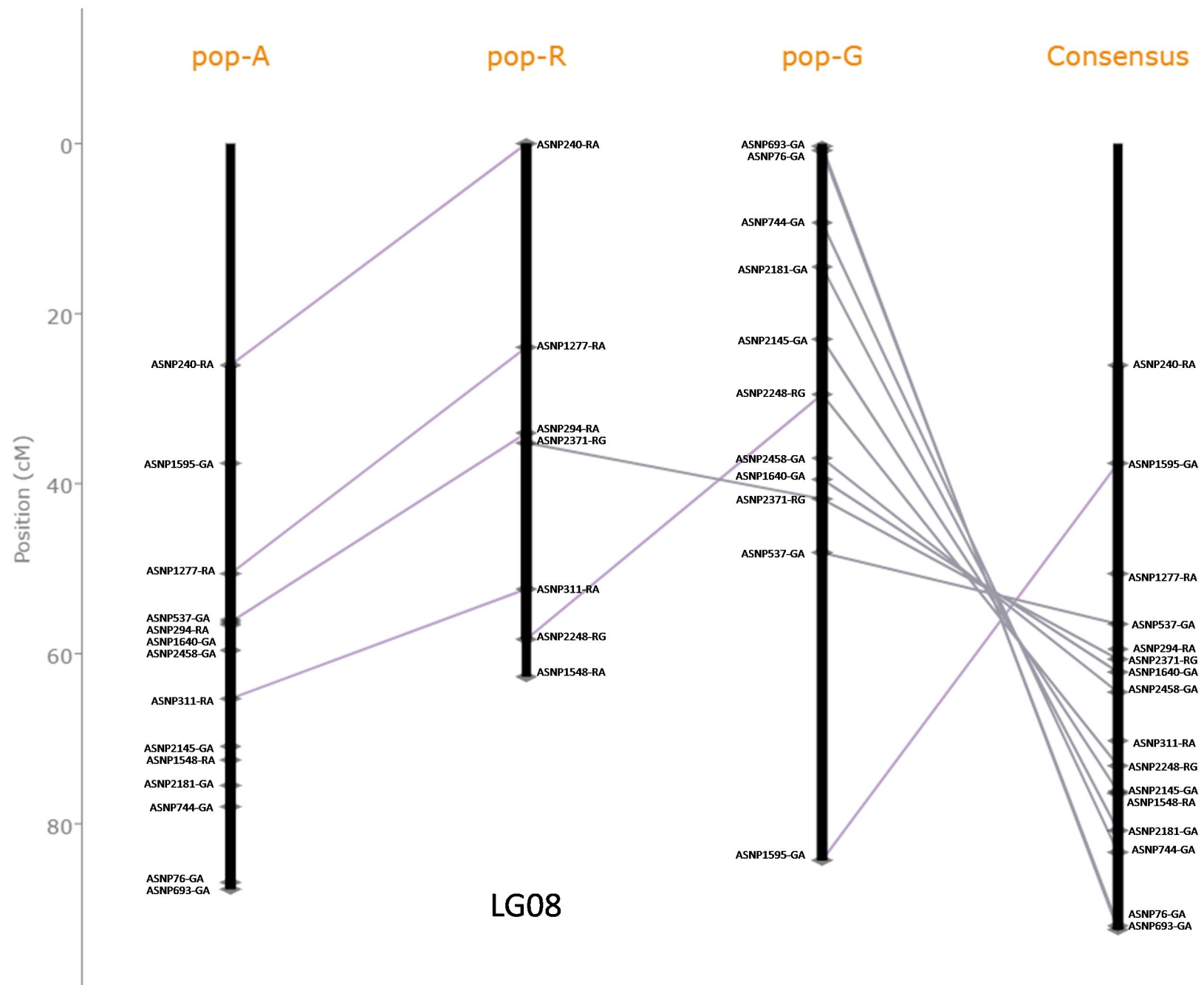

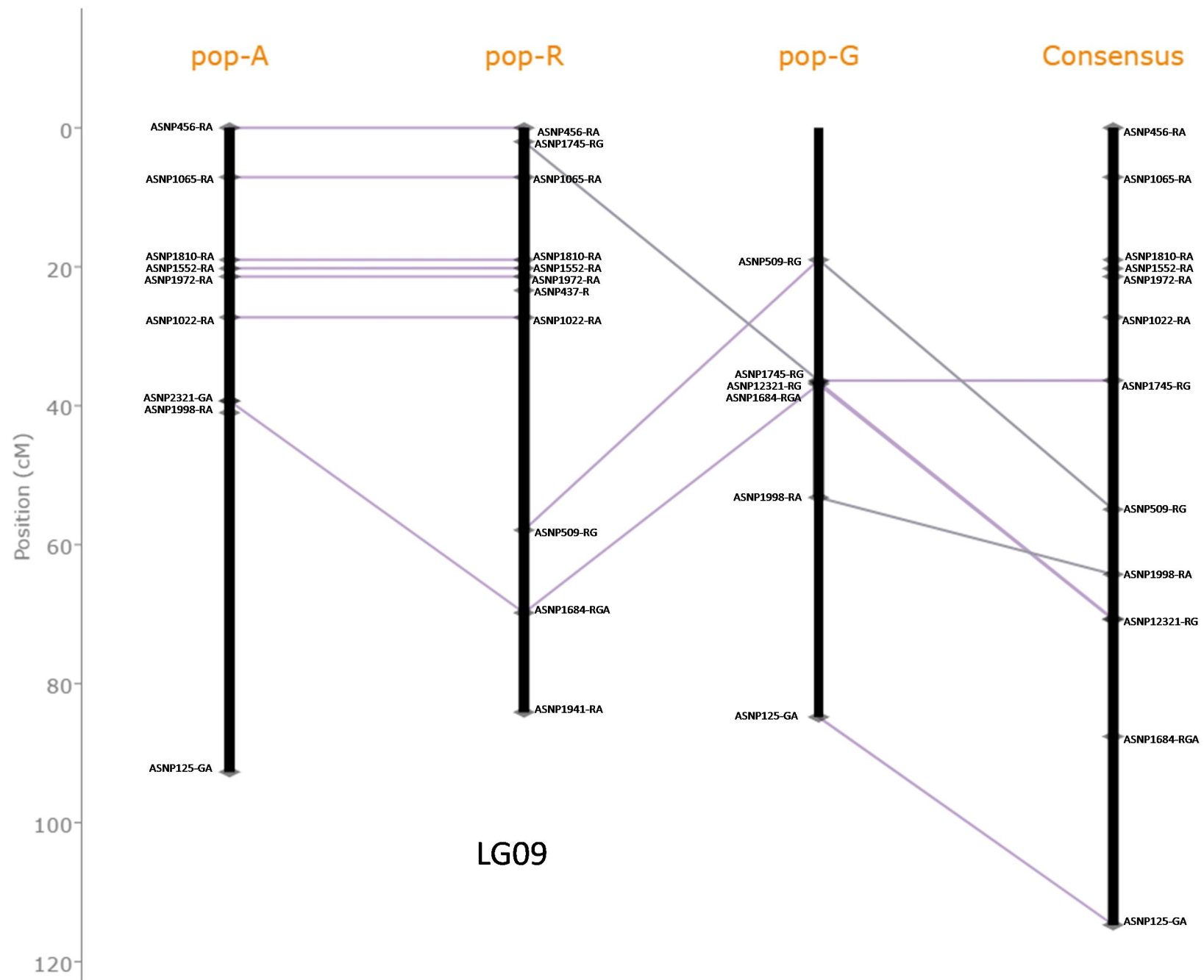

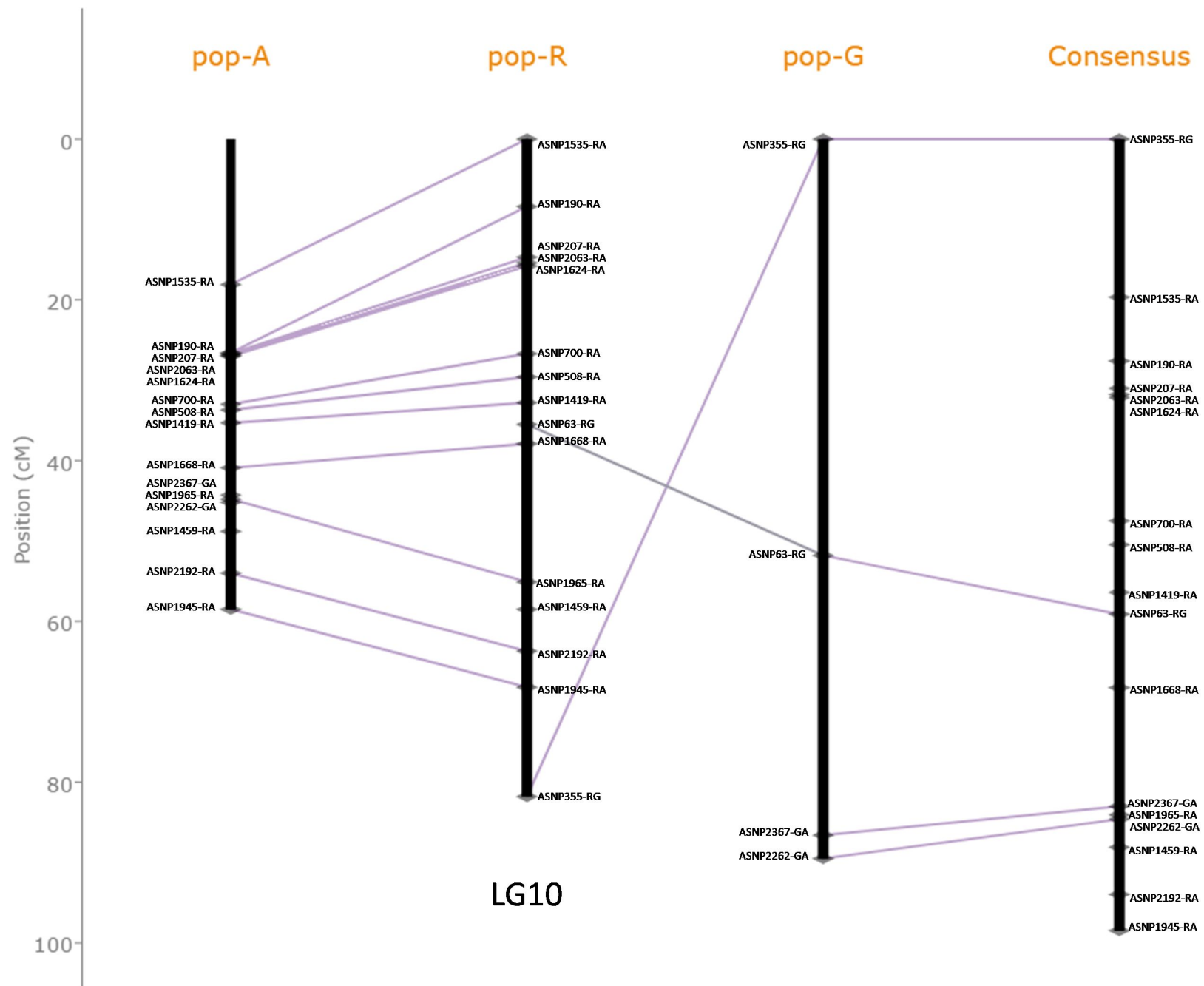

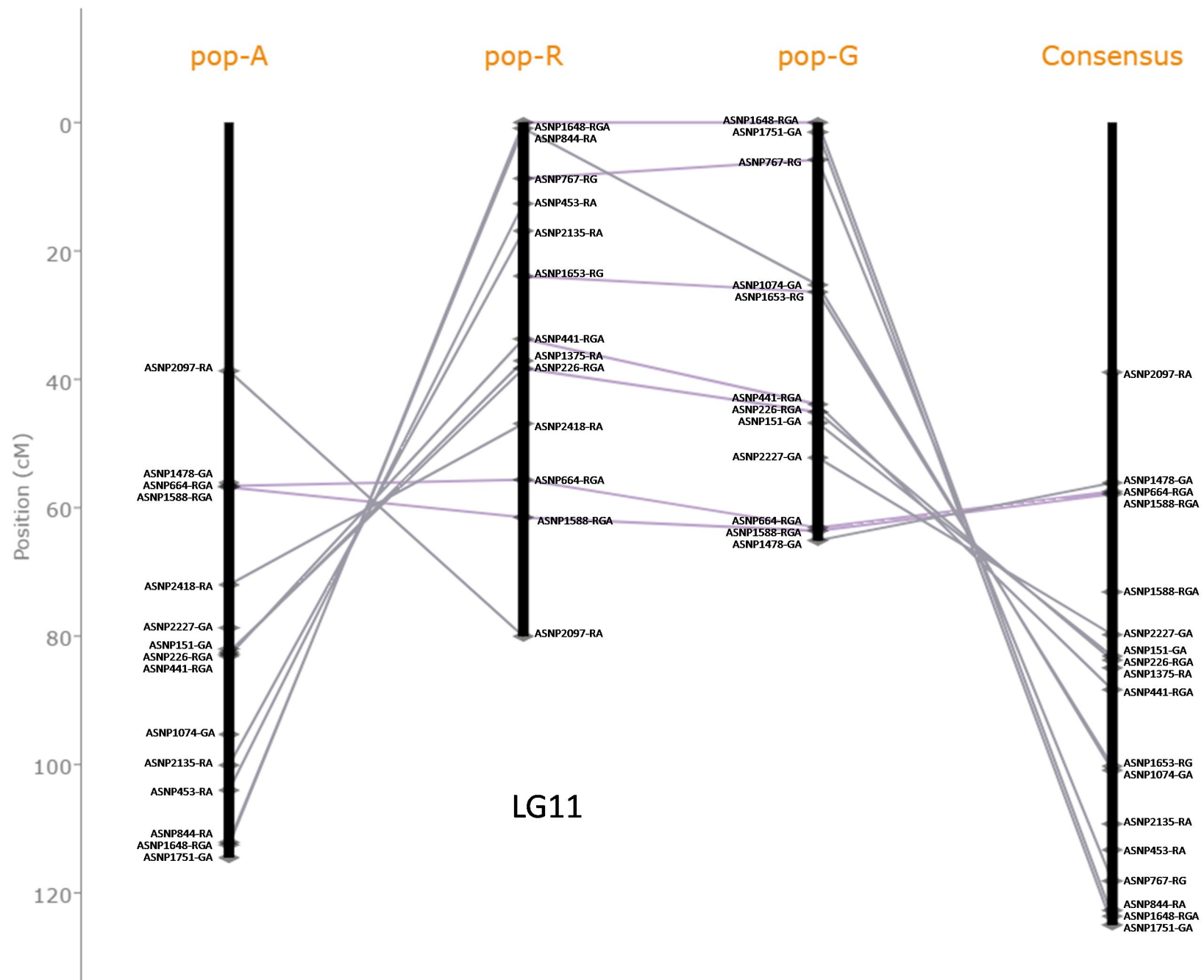

Supplement: S4 Fig — (PDF) [file pone.0179747.s006.pdf]
